# Redefining precision in biliary surgery: a paradigm shift from operative mastery to prognostic-driven strategy

**DOI:** 10.3389/fsurg.2026.1748054

**Published:** 2026-03-04

**Authors:** Zhi-Yuan Bai, Peng-Fei Zhang

**Affiliations:** 1 Department of Hepatobiliary Surgery, The First Hospital of Yulin, Yulin, China; 2 First Ward of General Surgery Department, The First Hospital of Yulin, Yulin, China

**Keywords:** biliary tract surgery, paradigm shift, patient-centered outcomes, precision medicine, prognosis-driven strategy, surgical precision

## Abstract

Traditional biliary surgery equated precision with technical mastery, focusing on flawless dissection and minimizing immediate complications. This article describes a fundamental paradigm shift in how surgical precision is defined. We argue that precision is evolving from technical execution to a comprehensive strategy driven by long-term patient outcomes. The analysis begins by examining limitations of the technique-centric approach. It then details the new paradigm's multidimensional aspects, including preoperative planning, intraoperative function preservation, and tailored postoperative care. The discussion addresses key technologies and conceptual innovations enabling this shift, concluding with future directions. Redefining precision is critically important. It guides personalized therapy, optimizes resource allocation, and advances biliary surgery toward improving long-term quality of life.

## 1 Introduction

### 1.1 Inherent challenges in biliary tract surgery

The biliary system serves as the liver's primary excretory pathway and presents a naturally complex surgical field due to its deep anatomical location, frequent anatomical variations, and close proximity to major blood vessels (1, 2). Its physiological role in bile metabolism and excretion is crucial for maintaining systemic homeostasis (2). Consequently, postoperative complications like bile leakage or anastomotic stricture can lead to long-term health issues that extend well beyond the immediate recovery period (3, 4). These complications often cause recurrent cholangitis, impair liver function, and significantly diminish patients' quality of life (3, 4). Therefore, biliary surgery is considered one of the most technically demanding subspecialties in general surgery, where outcomes are closely tied to the surgeon's skill.

### 1.2 Achievements and limitations of the traditional paradigm

In recent decades, the evolution of biliary surgery has centered on the paradigm of “technical mastery” (5). This philosophy equates surgical precision with intraoperative excellence, characterized by precise anatomical dissection, meticulous tissue handling, and flawless anastomotic technique (6). The advent of minimally invasive techniques, particularly the widespread adoption of laparoscopic and robotic platforms, represents the pinnacle of this paradigm (6). These technologies have reduced surgical trauma and improved short-term outcomes such as reduced blood loss, achieved through enhanced visualization and greater instrument stability (7). However, the limitations of this paradigm, which focuses primarily on “the success of the procedure itself,” have become increasingly apparent. An intense focus on overcoming technical challenges can overshadow the ultimate goal of therapy: the patient's long-term well-being (8). A technically perfect cholecystectomy or biliary anastomosis may still lead to poor long-term outcomes—including biliary dysfunction, disease recurrence, or diminished quality of life—if it fails to account for the patient's specific pathophysiology, need for long-term biliary patency, or oncological principles (9, 10). In essence, a “successful operation” does not automatically guarantee a “successful patient outcome”.

### 1.3 Proposing a paradigm shift

In light of these limitations, contemporary biliary surgery is undergoing a profound conceptual transformation—a paradigm shift from a surgeon-centric model of “technical precision” to a patient-outcome-oriented model of “prognosis-driven precision” (11, 12). This transition extends beyond the iterative improvement of technical tools; it represents a fundamental restructuring of treatment philosophy, clinical decision-making logic, and standards for evaluating efficacy in biliary surgery. Within this new paradigm, the concept of precision is significantly broadened and enriched. It requires broadening the perspective from the isolated surgical event to the entire continuum of patient care. Every aspect of care—from preoperative assessment and intraoperative strategy to postoperative management—must be guided by the overarching goal of optimizing long-term survival, functional preservation, and patient quality of life. This shift heralds a new, more mature, rational, and patient-centered era in biliary surgery.

## 2 Traditional paradigm: mastery of technique as the core of precision surgery

### 2.1 Core philosophy

The traditional paradigm in biliary surgery is fundamentally centered on a core philosophy: to perform operations that are technically perfect, minimally invasive, and aesthetically refined (11). In this framework, surgical precision was predominantly defined by the surgeon's technical skill and dexterity (11, 13). The primary goal was the complete removal of pathological tissue and flawless anatomical reconstruction, achieved through superior individual skill and experience (13). This approach viewed surgery as a blend of art and craft, emphasizing absolute control over each procedural step (14). Its ultimate ideal was to achieve an anatomically flawless and technically seamless procedure (15). Consequently, this paradigm profoundly influenced surgical training and culture, establishing technical skill refinement as the primary indicator of surgical proficiency (15).

### 2.2 Key dimensions

This philosophy was realized through three interconnected dimensions: The first was anatomical precision. This required a thorough understanding of the biliary system's complex anatomy and common variations, particularly in critical areas such as the Triangle of Calot and the hepatic hilum (16). The surgical objective involved clear exposure and unambiguous identification of target structures while ensuring absolute safety of adjacent blood vessels and organs (16, 17). This meticulous approach to dissection was fundamental for preventing intraoperative injuries.

The second dimension was technical precision. This focused on executing surgical maneuvers with exceptional skill, particularly in laparoscopic and robotic surgery, demonstrated through fine dissection using long instruments, precise suturing with minimal tension, and creating reliable anastomoses (6, 18). Technical precision directly affected the degree of tissue trauma, amount of blood loss, and quality of anastomotic healing (18, 19).

The third dimension involved optimization of perioperative metrics. In this paradigm, surgical quality was typically assessed using quantifiable short-term indicators, including operative duration, intraoperative blood loss, and rates of adverse events like bile duct injury (20, 21). These metrics collectively formed the immediate basis for determining a procedure's “success.”

### 2.3 Evaluation system

Guided by these dimensions, the traditional evaluation system primarily relied on intraoperative metrics and short-term complication rates. Common parameters included operative time, estimated blood loss, conversion-to-open rate, and complications within 30 postoperative days (such as surgical site infection, early bleeding, or bile leak) (22, 23). This system provided the advantages of objectivity, quantifiability, and suitability for comparative benchmarking, serving as the cornerstone for surgical quality assessment (24). However, its main limitation is its relatively short-term and narrow focus. While it effectively measures procedural success, it fails to adequately capture the long-term consequences of surgery for the patient (25). Outcomes such as durable biliary patency, long-term quality of life, and—for cancer patients—long-term survival rates are not fully captured (9, 10). Thus, while coherent within the traditional paradigm, this evaluation system is incomplete from the broader perspective of overall patient prognosis. To clearly illustrate the conceptual and practical differences between the traditional technique-driven paradigm and the emerging prognosis-driven strategy in biliary surgery, the key dimensions of this paradigm shift are summarized in Table 1.

**Table 1:** Evolution of surgical precision in biliary surgery: From technical mastery to prognosis-driven strategy.

Dimension	Traditional technique-driven precision	Prognosis-driven precision strategy
Core philosophy	Technical excellence and anatomical accuracy as the primary goal	Long-term survival, functional preservation, and quality of life as ultimate goals
Definition of precision	Precision equated with flawless operative execution	Precision defined as outcome-oriented decision-making across the entire care continuum
Preoperative focus	Imaging-based anatomical assessment and operability	Multidimensional risk stratification incorporating liver function, lymphatic involvement, tumor biology, and physiological reserve
Decision-making logic	“What operation can be performed safely?”	“What strategy optimizes the patient’s long-term prognosis?”
Intraoperative strategy	Anatomy-oriented resection with emphasis on complete removal	Function-oriented surgery prioritizing biliary continuity, liver preservation, and physiological integrity
Role of technology	Minimally invasive platforms to enhance dexterity and visualization	AI-assisted planning, radiomics, fluorescence imaging, navigation systems, and molecular guidance
Oncologic integration	Surgery considered the central curative modality	Surgery integrated with molecular profiling, neoadjuvant/adjuvant therapy, and immunotherapy
Postoperative management	Reactive complication management	Predictive risk modeling, tailored enhanced recovery, and structured long-term follow-up
Outcome evaluation metrics	Operative time, blood loss, short-term complication rates	Long-term biliary patency, recurrence risk, survival, patient-reported outcomes
Role of multidisciplinary care	Consultative and episodic	Continuous, strategy-defining multidisciplinary collaboration
Educational implication	Emphasis on manual skill acquisition	Emphasis on systems thinking, data interpretation, and prognostic reasoning
Value framework	Procedure-centered success	Value-based care balancing outcomes, costs, and patient benefit

## 3 New paradigm: a multidimensional, prognosis-driven strategy for precision

### 3.1 Core philosophy

**Figure 1:**
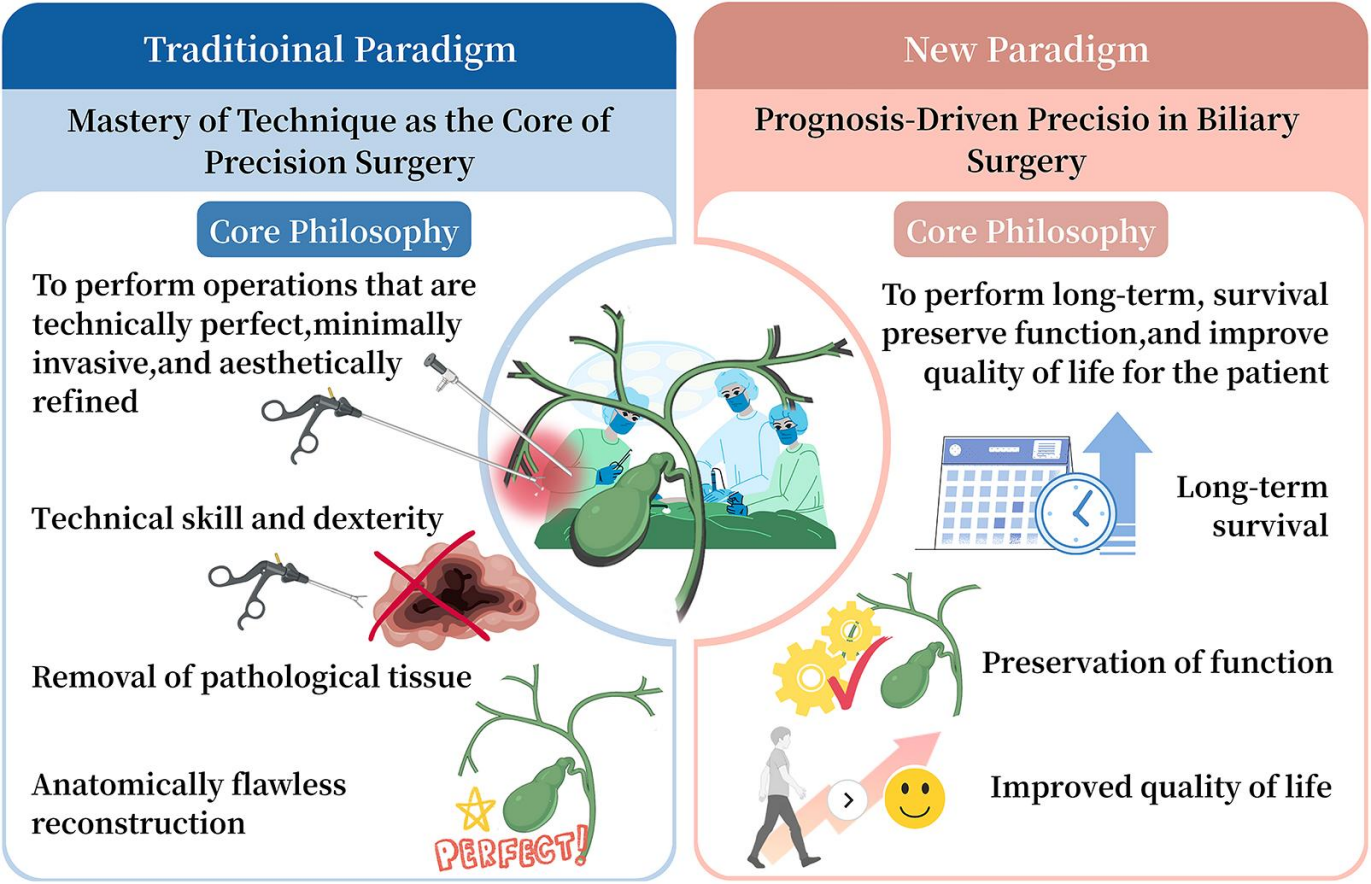
Traditional comparison of the traditional technique-centered paradigm and the new prognosis-driven paradigm in biliary surgery

The core philosophy of the emerging prognosis-driven paradigm is succinctly defined as “reverse-engineering the entire clinical pathway from the goal of optimal long-term patient outcomes” (26). This represents a fundamental shift in surgical logic, moving from asking “What operation can we perform?” to “What outcome does the patient need" (26)? Within this framework, the surgical procedure is no longer an isolated technical endpoint but a critical component of a comprehensive strategy designed to achieve specific long-term health goals (27). Every clinical decision, from initial diagnosis to final surgical plan, must serve the paramount goal of optimizing long-term survival, functional preservation, and quality of life (28). This philosophy places the patient's lifelong well-being at the center of care, requiring a holistic perspective from the surgical team that extends beyond the operating room (Figure 1).

### 3.2 The multidimensional aspects of the new paradigm

This philosophy translates into a system of multidimensional precision strategies spanning the entire patient journey, with a scope far broader than the traditional paradigm (Figure 1).

### 3.2.1 Precision in preoperative decision-making

The preoperative phase is transformed from mere surgical “preparation” into the starting point of precision therapy. First, this involves refined risk stratification and patient selection. This requires a multidimensional assessment beyond standard imaging, incorporating quantitative liver function tests, nutritional status screening, and evaluation of comorbidities and physiological reserve (29). Integrating this data enables precise identification of high-risk patients, predicts their tolerance for different surgical approaches, and informs optimal timing—or selection of less invasive palliative procedures when major resection is unsuitable (30). Second, it entails highly individualized surgical planning. Decision-making must be grounded in disease biology (30). For biliary cancers, this means incorporating tumor molecular subtype, grade, and stage to predict behavior, determining whether extended radical resection or a conservative, function-preserving approach is warranted (31). Strategies like the “liver-first” approach for hilar cholangiocarcinoma are based on precise calculation of future liver remnant volume and function to balance radicality with safety.

Preoperative assessment of lymphatic involvement is a critical yet frequently overlooked component of prognosis-based precision in biliary surgery (32, 33). In cholangiocarcinoma, regional and para-aortic lymph node status strongly correlates with systemic inflammatory burden, recurrence risk, and long-term survival (34). Incorporating high-resolution imaging, staging laparoscopy, and selective nodal sampling into preoperative workflows allows surgeons to differentiate patients who may benefit from radical resection from those better suited for non-surgical or palliative approaches (35). Prognostically, lymphatic tumor burden not only informs oncologic staging but also reflects host-tumor immune interactions that influence postoperative inflammatory responses and recovery trajectories (36).

#### 3.2.2 Precision in intraoperative strategy

Intraoperatively, the definition of precision expands from anatomical accuracy to functional preservation. The central tenet is a strategic shift from “anatomy-oriented” to “function-oriented” approach (37). The surgical objective is redefined as maximizing organ function and maintaining physiological pathways while eradicating disease (37, 38). For benign or low-grade malignant strictures, the goal shifts from simple resection to reconstructing physiological bile drainage continuity through techniques like biliaryplasty or duct-to-duct anastomosis, aiming for long-term functional cure (39).Technological advancements underpin this shift. Real-time imaging technologies like fluorescence guidance with indocyanine green (ICG) elevate the surgeon's view from static anatomy to dynamic functional anatomy (38). By visualizing liver segments and biliary excretion, ICG enables real-time definition of resection boundaries, assessment of tissue perfusion, and confirmation of biliary patency (38). This facilitates maximal preservation of functional liver units and guides precise bilio-enteric anastomosis placement, fundamentally reducing postoperative bile leak and stricture risk.

#### 3.2.3 Precision in postoperative management

Postoperative care is critical for securing excellent long-term outcomes, with precision manifested through proactive intervention and long-term planning. First is the integration of predictive medicine and enhanced recovery. Using specific surgical details and patient factors, predictive models prospectively identify high-risk patients for complications like bile leak or liver insufficiency, enabling targeted prevention (40, 41). Concurrently, enhanced recovery after surgery protocols are tailored to biliary patients to reduce surgical stress, shorten recovery, and improve outcomes through optimized pain management and early mobilization (42). Second is establishing systematic long-term follow-up and functional assessment. The new paradigm requires expanding follow-up beyond “recurrence-free status” to include long-term biliary patency, recurrent cholangitis incidence, liver function, and quality of life using standardized scales (9). This long-term data creates a crucial feedback loop that continuously validates and refines preoperative decisions and intraoperative strategies (9). This drives ongoing improvement of the entire clinical pathway, establishing a cycle where practice is consistently driven by long-term prognosis.

## 4 Key technologies and concepts driving the paradigm shift

The shift from a “mastery of technique” to a “prognosis-driven” paradigm is driven by the synergistic evolution of key technologies and core concepts (Figure 1).These elements provide the essential tools and frameworks for implementing this new approach.

### 4.1 Technology enablement

Advanced technologies form the foundation for prognosis-driven precision. First, artificial intelligence and radiomics are transforming preoperative planning (43). Using deep learning algorithms to analyze computed tomography or magnetic resonance imaging, these tools can automatically segment liver vessels and tumors, enabling virtual surgery simulations (43). This quantifies predicted future liver volume and function, providing data-driven support for safe surgical margins and elevating planning from empirical to individualized, objective strategy (44). Second, intraoperative navigation and augmented reality bridge the gap between preoperative plans and surgical execution (45). This technology superimposes patient-specific three-dimensional models onto the surgical field, providing visual guidance to locate tumors and identify critical structures in complex anatomy (45). This translates planning into precise execution, enhancing anatomical accuracy and safety.

For biliary tract cancers, molecular pathology and liquid biopsy are essential for enabling a truly personalized, biologically guided management continuum (46). Comprehensive molecular profiling has revealed distinct genetic and epigenetic subtypes of cholangiocarcinoma, including actionable alterations such as FGFR2 fusions and IDH1 mutations (47, 48). These molecular features enhance diagnostic accuracy and inform targeted therapeutic and immunotherapeutic strategies, which can improve survival in select patients (49, 50). Furthermore, this molecular stratification aids in surgical decision-making by identifying aggressive tumor subtypes that may benefit from neoadjuvant therapy prior to resection, thereby strategically integrating surgery into a tailored treatment pathway (47, 48, 51).

Complementing these clinical technologies, preclinical animal models are instrumental in advancing prognosis-driven management. They provide a vital platform for refining radiotherapy techniques through the precise evaluation of dose-volume relationships, radiation-induced liver injury, and compensatory hypertrophy within the future liver remnant (52–54). The insights derived from these models directly inform critical clinical applications, including radiotherapy planning, technical support system development, and preoperative volumetric assessment (55). Specifically, they enhance the treatment paradigm for patients undergoing neoadjuvant radiotherapy or multimodal therapy for cholangiocarcinoma. Consequently, findings from preclinical studies serve as a crucial link, connecting technical innovations with the overarching clinical goal of long-term functional organ preservation.

### 4.2 Conceptual innovation

Technology alone is insufficient without corresponding conceptual advances. The new paradigm equally depends on evolving core concepts. Essential is the routine practice of multidisciplinary team collaboration (56). The complexity of biliary diseases requires input from multiple specialties to determine optimal management. A standing multidisciplinary team with relevant specialists enables comprehensive assessment from diverse professional perspectives (56). This ensures diagnostic accuracy, appropriate timing, and optimized treatment strategies, serving as an institutional guarantee for best outcomes (56). Second, patient-reported outcomes must become a core evaluative standard. While traditional metrics remain important, they cannot fully capture treatment impact on patients' lives (26). Systematically collecting patient experiences through standardized questionnaires ensures medical decisions address patient needs, embodying patient-centered care (26). Ultimately, these efforts converge in value-based healthcare. This concept emphasizes that healthcare value comes from health outcomes relative to cost, not technical difficulty (57). This philosophy shifts focus from performing more procedures to performing the most beneficial ones, completing the transition from technique-oriented to prognosis-driven care.

## 5 Challenges and future directions

The prognosis-driven paradigm for precision biliary surgery shows great promise but faces several implementation challenges. Defining clear future directions is essential for overcoming these obstacles and advancing the field.

### 5.1 Prevailing challenges

First, technology access and the learning curve present major barriers. Advanced technologies like artificial intelligence and augmented reality involve high costs and complexity, hindering widespread adoption, especially in resource-limited settings (58). Additionally, surgeons and teams require substantial training to master these tools and adapt to new workflows (59). Second, data integration and standardization challenges limit the potential of big data. Prognosis-driven strategies depend on high-quality data from multiple sources (60). However, data from different institutions and systems often exist in different formats, creating “data silos” that complicate analysis (60). Third, transforming surgical education presents a fundamental challenge. Shifting surgical training from manual skills toward data interpretation, collaboration, and evidence-based decision-making requires profound educational reform (61). Finally, economic considerations are crucial. The technology and collaboration required by this paradigm increase initial costs (57). Therefore, health economics research must demonstrate long-term value through better outcomes and lower complication costs to secure support from policymakers.

### 5.2 Future directions

To address these challenges, several key directions are essential. First, “Digital Twin” technology could revolutionize preoperative planning (62). By creating personalized virtual models, surgeons can simulate procedures and optimize decisions before surgery (62). Second, advances in biomaterials and tissue engineering could transform biliary repair. New scaffold materials that promote tissue regeneration could address restenosis and achieve true functional restoration (63). Ultimately, the goal is to develop data-driven prognostic models. Integrating standardized data and using machine learning could provide quantitative predictions of individual patient outcomes for different treatments (64). This would support shared decision-making and enable truly personalized, prognosis-driven care.

### 5.3 Transplant oncology and immunosuppressive strategy optimization

In the emerging field of transplant oncology, the selection of immunosuppressive regimens plays a critical role in influencing tumor recurrence and disease-free survival (65, 66). Strategies that prioritize mTOR inhibitor–based protocols, immune monitoring, and personalized immunosuppression aim to balance graft protection with antitumor immune surveillance (66, 67). From a prognosis-driven perspective, optimizing immunosuppression is therefore not only a pharmacological consideration but also an essential component of long-term oncologic management for carefully selected patients with biliary malignancies undergoing transplantation (68, 69).

## 6 Summary

This article describes a fundamental transformation in biliary surgery: a shift from a “technical mastery” paradigm to a “prognosis-driven” one. The core of this shift is a redefinition of “precision” in biliary surgery. Precision is no longer limited to surgical skill and anatomical accuracy in the operating room. Instead, it now represents a strategic approach spanning the entire care pathway, consistently guided by the goal of long-term patient health and quality of life.

This redefinition of precision has important theoretical and practical implications. Conceptually, it marks biliary surgery's transition from technical prowess to a more mature, rational, and patient-centered discipline. This evolution requires future biliary surgeons to be more than technically skilled craftsmen focused solely on procedural success. They must become strategists capable of holistic oversight and evidence-based decision-making. Their ultimate goal is not just a perfect operation, but achieving the best possible long-term outcomes for each patient. Successfully implementing this paradigm will advance biliary surgery toward genuinely patient-centered care.

## Data Availability

The original contributions presented in the study are included in the article/Supplementary Material, further inquiries can be directed to the corresponding author/s.
